# Current trends, barriers, and facilitators of use of core outcome sets in Cochrane systematic reviews: Protocol

**DOI:** 10.12688/f1000research.133688.2

**Published:** 2023-09-25

**Authors:** Ian Saldanha, Karen Hughes, Susanna Dodd, Toby Lasserson, Jamie Kirkham, Samuel Lucas, Paula Williamson

**Affiliations:** 1 Department of Epidemiology, Johns Hopkins Bloomberg School of Public Health, Baltimore, MD, USA; 2 Department of Health Data Science, University of Liverpool, Liverpool, England, UK; 3 Central Editorial Unit, Cochrane, London, England, UK; 4 Centre for Biostatistics, The University of Manchester, Manchester, England, UK

**Keywords:** core outcome sets, systematic reviews, Cochrane, outcome choice, barriers, facilitators

## Abstract

**Background**: Core outcome sets (COS) represent agreed-upon minimum outcomes that should be reported in all studies in a given topic area. Cochrane reviews are considered among the most rigorously conducted systematic reviews (SRs). In 2019, seven of the first 100 published Cochrane SRs (7%) cited a COS in relation to choosing outcomes. A relevant COS existed but was not mentioned (or cited) for 27 of the remaining 93 SRs (29%). Among Cochrane Review Group editors surveyed in 2019, 86% felt that COS should definitely/possibly be used in Cochrane SRs. As of September 2019, the Cochrane Handbook recommends that SR teams consult resources that host relevant COS when choosing outcomes for the SR.

**Objectives**: (1) Examine the extent to which authors are currently considering COS to inform outcome choice in Cochrane protocols and completed SRs. (2) Understand author barriers and facilitators of using COS in Cochrane protocols and completed SRs.

**Methods:** We will examine the extent to which all Cochrane SRs published in the last 3 months of 2022 and all Cochrane protocols published in 2022: (a) cited a COS, (b) searched for COS, and (c) reported outcome inconsistency among included studies and/or noted the need for COS. One investigator will extract information from SRs and protocols; a second extractor will verify all information, discussing discrepancies to achieve consensus. Using Jisc Online Surveys ^®^, we will conduct an online anonymous survey of authors of all the included completed SRs and protocols to assess author awareness of COS and identify barriers and facilitators of using COS to inform outcome choice.

**Discussion:** This study will provide key information regarding uptake of COS by Cochrane SR authors and the barriers and facilitators that they experience. Our findings will inform approaches to increasing awareness and uptake of COS in future SRs, both with and beyond Cochrane.

## Background

Core outcome sets (COS) represent agreed-upon minimum outcomes that should be reported in all research studies in a given topic area. ^1^ COS are intended to increase the usefulness of research evidence and facilitate comparisons across studies. Although COS have traditionally been developed for use in clinical trials, increasingly COS are also developed for routine care or for registries. ^2^^,^^3^ A systematic review (SR) is a research effort whose goal is to identify and synthesize all relevant studies that fulfill pre-specified eligibility criteria to answer a specific research question(s). ^4^^,^^5^ Thus, systematic reviewers not only evaluate primary research that should have considered COS ^6^ but they should themselves also consider COS when choosing outcomes for the SR.

Cochrane SRs are considered among the most rigorously conducted SRs in the world. ^7^^–^^9^ We previously conducted an analysis and reported that only seven of the first 100 published Cochrane SRs in 2019 (7%) cited a COS in relation to choosing outcomes. ^10^ A relevant COS existed but was not mentioned (or cited) for 27 of the remaining 93 SRs (29%). For a further 6 reviews, a relevant COS was published after the protocol of the SR was developed. ^10^ We similarly conducted an analysis of all 67 comparative effectiveness SRs that were published between 2018 and 2020 by Evidence-based Practice Centers (EPCs) with funding from the U.S. Agency for Healthcare Research and Quality (AHRQ) (EPC SRs are also highly rigorous SRs). ^11^ We found relevant COS for 36 of the 67 EPC SRs (54%). ^11^ In the Cochrane analysis paper, we also reported results of a survey of editors of 36 (of the then 52) Cochrane Review Groups; most editors (31/36; 86%) felt that COS should definitely/possibly be used in Cochrane SRs. ^10^

Considerations regarding COS scope and contextual relevance to the SR notwithstanding, COS should be considered when choosing outcomes to be examined in SRs. ^11^ Use of existing COS represents an opportunity for systematic reviewers to leverage the efforts of COS developers in identifying outcomes for their SRs. Indeed, the 2 ^nd^ (i.e., most recent) edition of the Cochrane Handbook for Systematic Reviews of Interventions now recommends that SR teams consult resources that host relevant COS when choosing outcomes for the review. ^12^ However, this edition of the Handbook was first published in September 2019, which was after outcome choices were made for all SRs in the sample of Cochrane SRs published in 2019 that we analyzed. Therefore, we do not know the extent to which authors of recent Cochrane SRs (and protocols) are considering COS to inform choice of outcomes.

Use of COS to inform choice of outcomes can be considered a behavior. ^13^ In 2022, based on a survey of authors of clinical trials submitted to the top five medical journals (in terms of impact factor), Matvienko-Sikar and colleagues reported that the most common barrier to COS use was trialist preferences and choices regarding outcomes (68% of respondents), and the most common facilitator was trialist awareness and knowledge about COS (90%). ^14^ Also in 2022, Hughes and colleagues conducted qualitative interviews of clinical trialists in the UK and reported that the biggest barriers to COS uptake were trialist perceptions regarding COS characteristics (e.g., increased patient burden, COS being out of date) and the COS development process (e.g., the lack of inclusion of all relevant clinical specialties). ^15^ The biggest facilitators were trialist awareness and understanding of COS and funder and journal editor recommendations to use COS. ^15^

Outcomes chosen for trials and for SRs, even within the same topic area, have been shown sometimes to be inconsistent, perhaps reflecting differing priorities. ^16^^,^^17^ Moreover, for Cochrane SRs, these decisions are often shared between the authors and the editorial team (with input from the peer reviewers, including people with lived experience of the condition of interest). ^10^ It may be that these various parties have different priorities, although the SR authors are primarily responsible for choosing which outcomes to assess and this will likely influence the final set of outcomes for a Cochrane SR. The stages of protocol development and subsequent peer review help vet the choice of outcomes.

We are aware of two surveys assessing barriers to COS use by SR authors, specifically in the topic area of pain. ^18^^,^^19^ Boric and colleagues surveyed authors of SRs of interventions for neuropathic pain and reported that the main barrier to use of the Initiative on Methods, Measurement, and Pain Assessment in Clinical Trials (IMMPACT) COS was the lack of awareness of the full COS. ^18^ Similarly, Dosenovic and colleagues surveyed authors of SRs of interventions for postoperative pain in children and reported that the main barriers to use of the pediatric version of the IMMPACT COS (PedIMMPACT) were the lack of awareness, difficulties with implementation, and the lack of resources. ^19^

To our knowledge, the barriers and facilitators of use of COS have not been examined among Cochrane SR authors. In our previous analyses of Cochrane SRs and EPC SRs, we did not survey the authors. The insights that could be gained from such an examination would be crucial to inform approaches to increasing awareness and uptake of COS in future SRs, both within and beyond Cochrane.

## Objectives

This study has two objectives:

(1) Examine the extent to which authors are currently considering COS to inform outcome choice in Cochrane protocols and completed SRs.
(2) Understand author barriers and facilitators of using COS in Cochrane protocols and completed SRs.

## Methods

### Summary of methods

For Objective 1, we will conduct a cross-sectional analysis of recent Cochrane SRs to examine the extent to which they (a) cited a COS, (b) mentioned searching for COS, and (c) reported outcome inconsistency among included studies and/or noted the need for COS. For Objective 2, we will conduct an online survey of authors of these recent Cochrane SRs to identify barriers and facilitators of their using COS to inform outcome choice in SRs.

### Methods for Objective 1

Eligibility Criteria for Cochrane SRs: We will include all completed Cochrane SRs published in the last 3 months of 2022 and all Cochrane SR protocols published throughout 2022 that evaluate intervention effectiveness and/or harms. This period restriction will enable us to examine contemporary practices regarding outcome choice in Cochrane SRs. We will not restrict SRs or protocols by topic area or location of the authors or Cochrane Review Group. We will exclude SRs of qualitative studies, SRs of methodological topics, and SRs that only address prognosis, diagnostic accuracy, or etiology. A total of 294 SRs are eligible for Objective 1.

Identifying Cochrane SRs: We will identify relevant completed SRs and protocols by searching the *Cochrane Database of Systematic Reviews* through the Cochrane Library, restricting by the periods of interest.

Data extraction:

For all relevant SRs, we will extract information about whether the SR authors:

(1) *Cited* a COS in the context of choosing outcomes for the review, and
(2) *Mentioned having searched for* a COS to choose outcomes for the review.

For completed SRs only, we will extract information about whether the SR authors:

(3) *Noted any problems with outcome inconsistency* across the included studies and/or the need for outcome standardization/COS development.

For all SRs that cited or mentioned using a COS, and SRs that did not cite a COS but for which we found one, we will also extract:

(4) The extent to which the SR authors *used* outcomes from the COS. In other words, we will assess the extent of overlap between the COS outcomes and the SR outcomes using a framework that we developed ^11^ and has been used since then. ^20^ Briefly, we will focus on the outcome domains (the “what,” e.g., pain) but will not examine whether the “how” of the outcome (e.g., one instrument for measuring pain versus another) matched. The framework we developed considers matches to be *general* (i.e., nonspecific) or *specific.* Our approach to determining the type of match for pairs of outcomes is consistent with an approach that has been used previously. ^2^^,^^20^^,^^21^

For SRs that did not cite or mention having considered a COS (i.e., no to both #1 and #2 above), we will also extract whether:

(5) A relevant COS *exists.* We will do this by searching the Core Outcome Measures for Effectiveness Trials (COMET) database. Maintained by the COMET Initiative, this is a free, online, regularly updated, searchable database of COS. One investigator will assess the potential relevance of each identified COS to the topic of each SR following an approach used previously. ^10^^,^^20^^,^^22^ For identified COS, we will also extract information regarding when the COS was published. This will allow us to assess whether the COS was available during the stage of outcome choice for the SR/protocol.

For information extraction, we will review all sections of the SR/protocol report. One investigator will extract information from each SR/protocol and a second investigator will independently verify the extracted information.

Statistical analysis: We will calculate descriptive statistics (percentages and medians with 25 ^th^ and 75 ^th^ percentiles). We will conduct all data analyses using Stata Version 16 (College Station, Texas, USA).

### Methods for Objective 2

Eligibility Criteria for Cochrane SR authors: We will email a survey to the corresponding authors of all completed relevant Cochrane protocols and completed SRs from Objective 1. If a corresponding author does not respond to the survey or our email, we will send the survey to the senior (i.e., last) author. If similar nonresponse occurs, we will send the survey to another author from the author list (e.g., first author). At each request, we will suggest that the person(s) most familiar with the considerations during outcome choice for the SR complete a single survey for the SR.

Survey design and implementation: We will design and distribute the survey using Jisc Online Surveys ^®^ in English. We will send the author(s) of each relevant SR one of four versions of the survey:

• Version A: For SRs that cited/mentioned searching for a COS and the full COS was used.
• Version B: For SRs that cited/mentioned searching for a COS and some but not all COS outcomes were used.
• Version C: For SRs that did not cite/mention searching for a COS, but we identified a potentially relevant COS that could have been used.
• Version D: For SRs that did not cite/mention searching for a COS and we did not identify a potentially relevant COS that could have been used.

Items of interest for the surveys include:

1. Author process for outcome choice for the SR
2. Awareness of COS in general
3. Whether the authors searched for COS (regardless of whether the SR mentions that the authors searched for it)
4. Awareness of a relevant COS (if one exists for the SR)
5. Reasons for non-use of relevant COS (if one exists for the SR)
  o In instances where a COS exists but was not used, we will also ask authors about the factors that would influence their decision to incorporate the unused outcomes from the COS when completing the SR (for protocols) or when updating the review (for completed SRs).
6. Perceived barriers to COS use
7. Perceived facilitators of COS use.

For items 5, 6, and 7 above, **Box 1** lists examples of prespecified as well as open-ended response options.

Box 1. **Prespecified and open-ended response options for three specific items in the survey for Objective 2: reasons for non-use of existing COS, general barriers to COS use, and general facilitators of use of COS in SRs.**

**Table:** 

Reasons for non-use of existing COS • Did not know there was a COS. • COS target population was too narrowly/broadly defined for the SR population of interest. • COS target intervention was too narrowly/broadly defined for the SR intervention of interest. • COS was too old. • Other, specify: _____________ General barriers to use of COS in SRs. • Systematic reviewers’ preference to use their own outcomes • Inadequate knowledge about the existence of COS • Inadequate knowledge about how to use core outcomes • Poor quality and design of COS • Lack of involvement of systematic reviewers in COS development • Belief that primary study outcomes are different from SR outcomes • Other, specify: _____________ General facilitators of use of COS in SRs. • Use of COS can facilitate incorporating more studies into meta-analyses • Positive perceptions of COS • Good knowledge about the existence of COS • Good knowledge about how to use core outcomes • Availability of well-designed COS • Belief that primary study outcomes should be fundamentally akin to SR outcomes • Recommendation of COS use from SR funder • Recommendation of COS use from guideline developer who intended to use the SR findings • Other, specify: _____________

Institutional Review Board (IRB) approval: Before we distribute the surveys, we will obtain Research Ethics Committee approval from the University of Liverpool, UK.

Data analysis: We will analyze the data by calculating descriptive statistics. For open-ended responses to the survey, we will use content analysis. For overlapping questions across versions of the survey, we will analyze and report overall results as well as separate results by survey version. We will report information from the survey only in the aggregate.

### Dissemination of findings

We will disseminate the findings regarding both objectives of this study through publication of peer-reviewed manuscripts and presentation at international conferences, such as the Cochrane Colloquium.

### Study status

We have identified the Cochrane SRs and protocols and are currently extracting information for the study.

## Discussion

Use of COS in Cochrane SRs is important to improve outcome standardization, reduce research waste, and improve evidence synthesis regarding the effects of interventions in particular health areas. A possible limitation of the current study’s findings (if the survey response rate is poor) is that the findings may not be generalizable to all current Cochrane reviewers. However, it is expected that this study will provide useful findings regarding the extent of uptake of COS by Cochrane reviewers and key insights regarding author views and reasons for using, or not using, COS when deciding on outcomes for Cochrane SRs. The findings and insights are essential to better understand barriers and facilitators of COS uptake in Cochrane SRs, so that interventions to promote uptake can be developed and improved.

## Data Availability

No data are associated with this article.
