# Supplementary material for: Current trends, barriers, and facilitators of use of core outcome sets in Cochrane systematic reviews: Protocol
Source: F1000Res. 2023 Sep 25;12:735. Originally published 2023 Jun 23. [Version 2] doi: 10.12688/f1000research.133688.2 (PMC11468176; doi:10.12688/f1000research.133688.2)
Supplement: Peer review history

# Reviewer report: Reviewer response for version 2

**DOI:** 10.5256/f1000research.156385.r322214

**Published:** 2024-10-09

**Authors:** Simone Willis

**Affiliations:** 1 Cardiff School of Sport and Health Sciences, Cardiff Metropolitan University, Cardiff University, Cardiff, UK

This protocol presents a study that examines the use of core outcome sets (COS) in Cochrane Reviews and the barriers and facilitators for authors using COS in reviews. The authors aim to assess the extent to which COS are used in a set of Cochrane Reviews through searching and selecting studies from CDSR. This will be followed by a survey sent to authors of the relevant reviews about their use of COS and any barriers or facilitators to using COS. Overall, the protocol is written clearly and the study makes a worthwhile contribution.

**Rationale: **This section appropriately sets up the study and provides sufficient references to previous studies that have considered the use of COS in systematic reviews.

**Study design: **The study design is appropriate for addressing the objectives of the research. Identification process and review of studies is sufficient for addressing objective 1. Survey followed by content analysis is appropriate for addressing objective 2.

**Methods:** The methods are presented in a logical fashion. Considering objective 2, sufficient detail is presented in the methods for a protocol. For final publication, the full questionnaire should be presented.

 p. 4 “… we will assess the extent of overlap between the COS outcomes and the SR outcomes using a framework that we developed and has been used since then.”

 “Since then” is ambiguous, please specify in the text.

Is the study design appropriate for the research question?

Yes

Is the rationale for, and objectives of, the study clearly described?

Yes

Are sufficient details of the methods provided to allow replication by others?

Yes

Are the datasets clearly presented in a useable and accessible format?

Not applicable

Reviewer Expertise:

Systematic reviews

I confirm that I have read this submission and believe that I have an appropriate level of expertise to confirm that it is of an acceptable scientific standard.

# Reviewer report: Reviewer response for version 2

**DOI:** 10.5256/f1000research.156385.r209181

**Published:** 2024-09-17

**Authors:** Theresa Sipe

**Affiliations:** 1 Retired from Centers for Disease Control and Prevention, Atlanta, USA

I have reviewed the response from the authors. They have addressed my concerns and I have no further comments to make.

Is the study design appropriate for the research question?

Partly

Is the rationale for, and objectives of, the study clearly described?

Yes

Are sufficient details of the methods provided to allow replication by others?

Partly

Are the datasets clearly presented in a useable and accessible format?

Not applicable

Reviewer Expertise:

Methods for systematic reviews and efficacy reviews that identify individual evidence-based interventions, HIV prevention.

I confirm that I have read this submission and believe that I have an appropriate level of expertise to confirm that it is of an acceptable scientific standard.

# Reviewer report: Reviewer response for version 1

**DOI:** 10.5256/f1000research.146696.r191471

**Published:** 2023-08-31

**Authors:** Roger Chou

**Affiliations:** 1 Departments of Medicine, and Medical Informatics & Clinical Epidemiology, Oregon Health and Science University Foundation, Portland, Oregon, USA

This is a protocol for a study evaluating the extent to which Cochrane reviews utilize core outcomes sets, and to understand barriers and facilitators to use of core outcome sets through author surveys. The objectives and methods are clear and the study will provide interesting and useful information to inform future systematic reviews and guidance on reviews.

 Re: the methods, for reviews that don't use a core outcome set, the study basically focuses on whether a core outcome set is available. In cases where there is a core outcome set, I think it would be useful and perhaps more meaningful to also compare the degree to which the review evaluated outcomes in the core outcome set i.e. even if they don't cite a core outcome set they could have evaluated most or all of the outcomes.

 I also think it is important to consider that for some Cochrane review groups (e.g., Back and Neck) there is a set of outcomes that authors are asked to include. This is not a formal "core outcome set" but may be utilized like one (and these outcomes are often based on core outcome sets). In these cases I am not sure that not citing/using a core outcome set is necessarily problematic.

 In terms of barriers to use of core outcome sets, one thing that comes up is that Cochrane/GRADE restrict the number of outcomes to be evaluated. This can create issues when a core outcomes specifies many outcomes.

 Regarding reasons for not using core outcome sets (Box 1), some reviews could be focused on a particular outcome e.g. a particular harm.

 In the Methods would be useful to describe the number of reviews/authors that are expected to be included, to give some sense of how robust the data and surveys will be.

Is the study design appropriate for the research question?

Partly

Is the rationale for, and objectives of, the study clearly described?

Yes

Are sufficient details of the methods provided to allow replication by others?

Yes

Are the datasets clearly presented in a useable and accessible format?

Not applicable

Reviewer Expertise:

Systematic reviews and systematic review methods

I confirm that I have read this submission and believe that I have an appropriate level of expertise to confirm that it is of an acceptable scientific standard, however I have significant reservations, as outlined above.

# Response

**Published:** 2023-09-20

**Authors:** Ian Saldanha

**Affiliations:** Epidemiology, Johns Hopkins University, Baltimore, Maryland, USA

*This is a protocol for a study evaluating the extent to which Cochrane reviews utilize core outcomes sets, and to understand barriers and facilitators to use of core outcome sets through author surveys. The objectives and methods are clear and the study will provide interesting and useful information to inform future systematic reviews and guidance on reviews.*

Response: We thank Dr. Chou for reviewing this manuscript and providing useful comments.

*Re: the methods, for reviews that don't use a core outcome set, the study basically focuses on whether a core outcome set is available. In cases where there is a core outcome set, I think it would be useful and perhaps more meaningful to also compare the degree to which the review evaluated outcomes in the core outcome set i.e. even if they don't cite a core outcome set they could have evaluated most or all of the outcomes.*

Response: We believe in the second sentence of this comment, Dr. Chou is referring to cases where there isn’t a core outcome set cited. We agree with the suggestion and have modified the text in the Data Extraction section of the Methods accordingly.

*I also think it is important to consider that for some Cochrane review groups (e.g., Back and Neck) there is a set of outcomes that authors are asked to include. This is not a formal "core outcome set" but may be utilized like one (and these outcomes are often based on core outcome sets). In these cases I am not sure that not citing/using a core outcome set is necessarily problematic.*

Response: We agree that this is likely the case for some Cochrane review groups. We anticipate that this sentiment will come through during the surveys when we ask authors what influenced the choice of outcomes for the SRs. Regardless, we believe that whether core outcome sets are used or not used should be clearly reported. Searching for core outcome sets is in keeping with current guidance in the Cochrane Handbook for Systematic Reviews of Interventions.

*In terms of barriers to use of core outcome sets, one thing that comes up is that Cochrane/GRADE restrict the number of outcomes to be evaluated. This can create issues when a core outcomes specifies many outcomes.*

Response: We agree that this may be the case for some core outcome sets but not most (the average number of outcomes in core outcome sets is about 6). It will be of interest to see if this is stated as an ‘other’ barrier by survey respondents.

*Regarding reasons for not using core outcome sets (Box 1), some reviews could be focused on a particular outcome e.g. a particular harm.*

Response: We don’t agree that being made to include a particular harm precludes a systematic review author from considering core outcome sets. By definition, a core outcome set is a minimum set of outcomes. Systematic reviewers should feel free to include additional outcomes. But, to Dr. Chou’s earlier comment, there may be restrictions to the number of outcomes in the systematic review.

*In the Methods would be useful to describe the number of reviews/authors that are expected to be included, to give some sense of how robust the data and surveys will be.*

Response: We have inserted the following sentence at the end of the Eligibility Criteria for Cochrane SRs section of the Methods: “A total of 294 SRs are eligible for Objective 1.”

# Reviewer report: Reviewer response for version 1

**DOI:** 10.5256/f1000research.146696.r194904

**Published:** 2023-08-29

**Authors:** Theresa Sipe

**Affiliations:** 1 Retired from Centers for Disease Control and Prevention, Atlanta, USA

This protocol entitled ‘Current trends, barriers, and facilitators of use of core outcome sets in Cochrane systematic reviews: Protocol’  is describing a protocol of a study that will examine systematic review (SR) protocols and completed reviews for inclusion of core outcome sets (COS) as well as conduct a survey of SR protocol/review authors for barriers and facilitators of using COS.

 In general, the protocol is well written and thorough in descriptions. There is some repetition and long sentences that could be reduced. The background does a good job explaining COS and some additional context will help readers not familiar with this concept.

 Background, p. 3 of 8, 1 ^st^ paragraph. Term ‘evidence ecosystem’ needs explanation. Seems that time context is needed. The time context will help with understanding the study done in 2019 that is cited in next paragraph.  

 2 ^nd^ paragraph – 1 ^st^ sentence needs citation.  

 3 ^rd^ paragraph – 1 ^st^ sentence is long and has a double negative, making it confusing. It also is a conclusion statement without evidence. Encouragement to do the action may be a better way to word the sentence or re-word to become common practice. 2 ^nd^ sentence - ‘(i.e., most recent)’ may not be necessary. Last sentence is unclear and Cochrane SRs are personified. It would be the extent in which authors of Cochrane SRs/protocols are considering COS.

 7 ^th^ paragraph, 1 ^st^ sentence – the text in parentheses is long and can be shortened ‘(in our previous analyses, we did not survey the authors…)’. Last sentence should ‘both with and beyond Cochrane’ be ‘both within and beyond Cochrane’? If so, this fix is needed for the abstract. 

 Methods p. 4 of 8. The design of the study is not stated. It seems to have systematic review components (literature search, inclusion/exclusion criteria, data abstraction) but does not meet all of the requirements for a systematic review. However, it could be considered a rapid review. Regardless, please state what the study design is.

 Methods for Objective 1, p. 4 of 8,  eligibility criteria -  Will there be any exclusions?

 (4) ‘since’ at the end of the sentence may not be grammatically correct.

 Statistical analyses, p. 4 of 8 – the authors stat that medians and IQRs will be used, however this term is often misused. IQRs are a single number (75 ^th^ percentile minus 25 ^th^ percentile). So maybe the authors mean ‘interquartile interval’ or more simply 25 ^th^ and 75 ^th^ percentiles.

 Eligibility Criteria for Cochran SR authors, p. 5 of 8 – the summary of methods on p. 4 says that the surveys are anonymous. How will the nonresponse be known if the surveys are anonymous?

 Survey design and implementation, p. 5 of 8 - ‘complete a single response for the SR’. It may be more clear to say ‘complete a single survey for the SR’ so as not to confuse with responses to questions on the survey. Will there be a pilot test of the survey?

 Items of interest for the surveys include, p. 5 of 8. #5: Reasons for non-use of relevant COS. The sub-bullet stats that the researchers ‘will ask authors whether they will consider incorporating the unused outcomes from COS when completing the SRs…’. This is not an objective of the paper. It seems to be putting the respondents in an awkward position and could lead to socially desirable responses. There may be many reasons why authors cannot add the COS at this time. Perhaps changing to an intention question that asks how likely they would be to add COS in the future would be better. Also, add this component to the objectives if needed. 

 Box 1, p. 6 of 8 – Suggest making all bulleted items match grammatically. For example, some bullets have verbs and some do not. For the next to last bullet about Guideline developer: the text in parentheses is confusing. Also it could be made broader than Guideline developer as not all SRs are used for guidelines.

 Discussion, p. 6 of 8 – Please add something about expected limitations of the evidence.

 Note: The peer review of this protocol is that of the reviewer and does not necessarily represent the official position of the Centers for Disease Control and Prevention.

Is the study design appropriate for the research question?

Partly

Is the rationale for, and objectives of, the study clearly described?

Yes

Are sufficient details of the methods provided to allow replication by others?

Partly

Are the datasets clearly presented in a useable and accessible format?

Not applicable

Reviewer Expertise:

Methods for systematic reviews and efficacy reviews that identify individual evidence-based interventions, HIV prevention.

I confirm that I have read this submission and believe that I have an appropriate level of expertise to confirm that it is of an acceptable scientific standard, however I have significant reservations, as outlined above.

# Response

**Published:** 2023-09-20

**Authors:** Ian Saldanha

**Affiliations:** Epidemiology, Johns Hopkins University, Baltimore, Maryland, USA

*This protocol entitled ‘Current trends, barriers, and facilitators of use of core outcome sets in Cochrane systematic reviews: Protocol’  is describing a protocol of a study that will examine systematic review (SR) protocols and completed reviews for inclusion of core outcome sets (COS) as well as conduct a survey of SR protocol/review authors for barriers and facilitators of using COS.*

*In general, the protocol is well written and thorough in descriptions. There is some repetition and long sentences that could be reduced. The background does a good job explaining COS and some additional context will help readers not familiar with this concept.*

Response: We thank Dr. Sipe for reviewing this manuscript and for providing useful comments. We have edited the manuscript and responded to the comments below.

*Background, p. 3 of 8, 1st* *paragraph. Term ‘evidence ecosystem’ needs explanation. Seems that time context is needed. The time context will help with understanding the study done in 2019 that is cited in next paragraph.* 

Response: We have removed reference to the evidence ecosystem. Instead, we specifically note the other targets of core outcome sets (i.e., for routine care and for registries).

*2nd* *paragraph – 1st* *sentence needs citation.*  

Response: We have added references to support the first sentence.

*3rd* *paragraph – 1st* *sentence is long and has a double negative, making it confusing. It also is a conclusion statement without evidence. Encouragement to do the action may be a better way to word the sentence or re-word to become common practice. 2nd* *sentence - ‘(i.e., most recent)’ may not be necessary. Last sentence is unclear and Cochrane SRs are personified. It would be the extent in which authors of Cochrane SRs/protocols are considering COS.*

Response: We have reworded the first sentence to remove the double-negative phrasing. We have retained the “most recent” in the second sentence because it illustrates the currency of the guidance from Cochrane and because it was released after our prior work, which is discussed in the previous paragraph. We have clarified the last sentence as suggested.

*7th paragraph, 1st sentence – the text in parentheses is long and can be shortened ‘(in our previous analyses, we did not survey the authors…)’. Last sentence should ‘both with and beyond Cochrane’ be ‘both within and beyond Cochrane’? If so, this fix is needed for the abstract.* 

Response: In the first sentence, we have removed the parenthetical text to be a full sentence and reworded it for clarity. We have made the suggested fix to the last sentence in paragraph 7 as well as in the Abstract.

*Methods p. 4 of 8. The design of the study is not stated. It seems to have systematic review components (literature search, inclusion/exclusion criteria, data abstraction) but does not meet all of the requirements for a systematic review. However, it could be considered a rapid review. Regardless, please state what the study design is.*

Response: We have added a design descriptor for Objective 1 (“cross-sectional analysis”). The text for Objective 2 includes a design descriptor (“online survey”).

*Methods for Objective 1, p. 4 of 8,  eligibility criteria -  Will there be any exclusions?*

Response: We have added the following exclusions: “We will exclude SRs of qualitative studies, SRs of methodological topics, and SRs that only address prognosis, diagnostic accuracy, or etiology.”

*(4) ‘since’ at the end of the sentence may not be grammatically correct.*

Response: We have inserted the word “then” after “since”.

*Statistical analyses, p. 4 of 8 – the authors stat that medians and IQRs will be used, however this term is often misused. IQRs are a single number (75th* *percentile minus 25th* *percentile). So maybe the authors mean ‘interquartile interval’ or more simply 25th* *and 75th* *percentiles.*

Response: We have replaced IQRs with “25 ^th^ and 75 ^th^ percentiles”.

*Eligibility Criteria for Cochran SR authors, p. 5 of 8 – the summary of methods on p. 4 says that the surveys are anonymous. How will the nonresponse be known if the surveys are anonymous?*

Response: We have removed the word “anonymous” throughout the manuscript.

*Survey design and implementation, p. 5 of 8 - ‘complete a single response for the SR’. It may be more clear to say ‘complete a single survey for the SR’ so as not to confuse with responses to questions on the survey. Will there be a pilot test of the survey?*

Response: We have made this change.

*Items of interest for the surveys include, p. 5 of 8. #5: Reasons for non-use of relevant COS. The sub-bullet stats that the researchers ‘will ask authors whether they will consider incorporating the unused outcomes from COS when completing the SRs…’. This is not an objective of the paper. It seems to be putting the respondents in an awkward position and could lead to socially desirable responses. There may be many reasons why authors cannot add the COS at this time. Perhaps changing to an intention question that asks how likely they would be to add COS in the future would be better. Also, add this component to the objectives if needed.* 

Response: We have modified the sentence to indicate that we will ask authors of such SRs about the factors that would influence their decision to incorporate the unused outcomes from the COS in future SRs.

*Box 1, p. 6 of 8 – Suggest making all bulleted items match grammatically. For example, some bullets have verbs and some do not. For the next to last bullet about Guideline developer: the text in parentheses is confusing. Also it could be made broader than Guideline developer as not all SRs are used for guidelines.*

Response: We have made the text in the bullet items match grammatically. We have edited the guideline developer text to state “Recommendation of COS use from guideline developer who intended to use the SR findings”.

*Discussion, p. 6 of 8 – Please add something about expected limitations of the evidence.*

Response: We have added the following limitation to the Discussion section: “A possible limitation of the current study’s findings (if the survey response rate is poor) is that the findings may not be generalizable to all current Cochrane reviewers.”
